# The antioxidant activity of *Beta vulgaris* leaf extract in improving scopolamine-induced spatial memory disorders in rats

**Published:** 2017

**Authors:** Shadieh Hajihosseini, Mahbubeh Setorki, Zahra hooshmandi

**Affiliations:** 1 *Department of Biology, Sanandaj Branch, Islamic Azad University, Sanandaj ,Iran*; 2 *Department of Biology, Izeh Branch, Islamic Azad University, Izeh, Iran*

**Keywords:** B. vulgaris, Leaf extract, Antioxidant activity, Spatial memory

## Abstract

**Objective:**

Medicinal plants have attracted global attention due to their safety as well as their considerable antioxidant content that helps to prevent or ameliorate various disorders including memory impairments. This study was conducted to investigate the effect of beet root (*Beta vulgaris*) leaf extract on scopolamine-induced spatial memory impairments in male Wistar rats.

**Materials and Methods:**

Male Wistar rats were randomly divided into 5 groups (n=10): Control (C), scopolamine 1 mg/kg/day (S), scopolamine+50 mg/kg *B. vulgaris* leaf extract (S+B 50), scopolamine+100 mg/kg *B. vulgaris* leaf extract (S+B 100) and scopolamine+200 mg/kg *B. vulgaris* leaf extract (S+B 200). Morris water maze task was used to assess spatial memory. Serum antioxidant capacity and malondialdehyde (MDA) level were also measured.

**Results:**

Group S spent significantly less time in the target quadrant compared to the control group, and the administration of *B. vulgaris* leaf extract (100 and 200 mg/kg) significantly increased this time (p<0.05). Scopolamine decreased serum antioxidant capacity and increased serum MDA level yet insignificantly. *B. vulgaris* extract (200 mg/kg) significantly increased the antioxidant capacity and decreased serum MDA level in scopolamine-treated rats (p<0.05).

**Conclusion:**

Our results suggested that *B. vulgaris* leaf extract could ameliorate the memory impairments and exhibited protective effects against scopolamine-induced oxidation. Further investigation is needed to isolate specific antioxidant compounds from *B. vulgaris* leaf extract with protective effect against brain and memory impairments.

## Introduction

Learning and memory play key roles in daily human life. Learning is the basis of all teaching and training programs. By identifying compounds that can improve these two processes, mental and memory status can be greatly improved. Studies showed that many drugs can affect memory and learning, for example, cholinergic drugs have a positive effect on memory, while anticholinergic and anesthetic drugs have negative effects on memory (Uttara et al., 2007 ▶; Moopanar et al., 2005 ▶; Hosseini et al., 2015 ▶). Scopolamine is an anticholinergic drug that blocks the action of acetylcholine within the nervous system. It has also been shown that scopolamine-induced cholinergic blockade induces significant memory impairment. Scopolamine administration is also associated with enhanced brain oxidative stress (Warner et al., 2004 ▶).

High rate of reactive oxygen species and free radicals production due to oxidative stress can lead to peroxidation of biomolecules such as lipids, proteins and damage to DNA that cause memory impairments (Sulakhiya et al., 2016 ▶) Brain tissue is more sensitive to the effect of free radicals due to its high oxygen demands, abundant lipid content, and relative paucity of antioxidant enzymes compared to other organs (Rasoolijazi et al ▶., Vincent et al., 2004 ▶).

The use of medicinal plants with high levels of antioxidant components is one of the most efficient ways to minimize the adverse effects of free radicals and treat diseases. While synthetic drugs may be unsafe for human and the environment, medicinal plants and their derivatives are growingly accepted worldwide due to their safety (Sulakhiya et al., 2016 ▶; Zaidi et al., 2014 ▶). In this regard, it has been frequently shown that antioxidant agents reverse scopolamine-induced memory impairments (Hosseini et al., 2015 ▶; Yang et al., 2009 ▶). 

Beetroot (*Beta vulgaris*) is a plant from the family Chenopodiaceae. *B. vulgaris* is native to Mediterranean regions and is extensively cultivated in different countries including Iran (Sulakhiya et al., 2016 ▶). Roots and leaves of *B*.* vulgaris* are used in traditional medicine, to treat different diseases. *B*. *vulgaris* leaves have diuretic, purgative, laxative, and aphrodisiac activity (Sulakhiya et al., 2016 ▶*;* Jain et al., 2011 ▶*;* El Gamal et al., 2014 ▶*). B*.* vulgaris* leaves contain various phytoconstituents such as betalains, flavonoids, polyphenols, vitamins, and minerals (Sulakhiya et al., 2016 ▶; Jain et al., 2012 ▶; Bolkent et al., 2007 ▶; Mroczek et al., 2012 ▶)*. B*.* vulgaris* leaves have antioxidant, anticancer, hepatoprotective, nephroprotective, wound healing, and anti‑inflammatory activities (Sulakhiya et al., 2016 ▶; El Gamal et al., 2014 ▶; Babu et al., 2010 ▶; Jain et al., 2011 ▶; Sacan et al., 2010 ▶), but its potential neuroprotective effects have not yet been tested.

Considering the important role of reactive oxygen species and free radicals in brain oxidative damage and memory impairments as well as the antioxidant potential of *B*.* vulgaris* leaf extract, this study was conducted to evaluate the effect of *B*. *vulgaris* leaf extract on scopolamine-induced brain tissue damage in rats. 

## Materials and Methods

**Preparation of ethanolic** ***B. vulgaris*** **leaf extract**

Fresh *B*.* vulgaris *leaves were shade-dried, pulverized, and macerated with 97% ethanol for 5 hr. The resulting extract was then filtered, and the filtrate was concentrated and dried using vacuum distillation at 40°C (Al-Jassabi et al., 2013 ▶)

**Determination of antioxidant activity of** ***B. vulgaris*** **leaf extract**

Briefly, 2 ml of *B. vulgaris* leaf extract or butylated hydroxytoluene (BHT) at different concentrations (prepared in ethanol) were added to 2 mL of DPPH solution (0.1 Mm in ethanol). After 15 min at room temperature in the dark, the absorbance of samples were measured at 517 nm wavelength. The mixture of ethanol (2 mL) and DPPH solution (2 mL) served as control. The scavenging activity percentage was determined by the following equation (Rabiei et al., 2014 ▶)

(AA%) = 100 × (A_control_-A_sample_)/A_control_

AA; scavenging activity

A_control_: absorbance of control

A _samples_: absorbance of samples

**Determination of total phenolic content of** ***B. vulgaris*** **leaf extract**

Briefly, 0.1 ml of diluted extract (0.01 g in 10 ml of 60°C methanol) was mixed with 0.5 ml of Folin-Ciocalteu reagent. After 3-5 min, 0.4 mL of 7.5% sodium carbonate solution was added to the mixture and left at room temperature for 30 min. The mixture absorbance was measured at 750 nm wavelength against distilled water blank. A standard calibration curve was plotted using different concentrations of gallic acid. The phenolic content was expressed as “mg gallic acid equivalents (GAEs)/g of the sample” (Rabiei et al., 2014 ▶).

**Determination of total flavonoid contents of** ***B. vulgaris*** **leaf extract**

The total flavonoid content of *B. vulgaris *leaf extract was determined using colorimetric method. The diluted extract (0.5 ml) was mixed with 0.5 ml of 2% aluminum chloride and 3 ml of 5% potassium acetate. After 40 min of incubation at room temperature, the absorbance of the reaction mixture was measured at 415 nm wavelength. A standard curve was prepared using different concentrations of rutin solution. The total flavonoid content was expressed as “mg of rutin equivalents/g of dried extract” by using a standard curve of rutin (Rabiei et al., 2014 ▶).

**Determination of total flavonolic content of** ***B. vulgaris*** **leaf extract**

The diluted extract (0.1 ml) was mixed with 0.5 ml of 2% aluminum chloride and 3 ml of 5% potassium acetate and left at room temperature for 2.5 hr. After incubation, the mixture absorbance was measured at 415 nm wavelength. Total flavonolic content was expressed in terms of rutin equivalent (mg/g), which is a common reference compound (Rabiei et al., 2014 ▶).

**Animals and treatments**

Adult male Wistar rats (weighing 200–250 g) were kept in experimental animal care center of Islamic Azad University of Sanandaj, Sanandaj, Iran. Rats were maintained under standard laboratory conditions of 25 ± 2°C temperature and 55 ± 5% humidity with 12 hr light-dark cycle with free access to water and standard laboratory food. The rats were randomly divided into 5 groups of 10 each. Control group (C) received distilled water alone intraperitoneally (ip); scopolamine-treated group (S) received distilled water 30 min after scopolamine injection (1 mg/kg/day, ip); Extract-treated groups received *B. vulgaris *leaf extract (50, 100, and 200 mg/kg/day, ip) 30 min after scopolamine injection for 14 days. The doses of scopolamine and *B. vulgaris* extract were selected based on the previous studies (Hosseini et al., 2015 ▶; Sulakhiya et al., 2016 ▶)

After treatment, spatial memory task was performed using Morris water maze. Then, the animals were put under deep anesthesia using chloral hydrate and then, cardiac blood samples were collected. The blood samples were centrifuged and their serum was isolated. Then, the biochemical analysis was carried out.

**Spatial memory**

To evaluate spatial memory, the rats were tested in Morris water maze which is a black circular tank with a diameter of 136 cm and height of 60 cm high, filled with 24 ± 1°C water to a depth of 25 cm. A hidden circular platform (diameter: 10 cm) was located in the center of the Southwest quadrant and submerged 1 cm below the surface of the water. The maze was located in a room containing many visual cues such as bookshelves, refrigerator, and poster. Each rat experienced two sessions of four trials per day for 5 consecutive days. During each trial, the rats were individually placed in the pool and released in the release positions randomly determined for each trial by computer. The rats were allowed to swim until they found the platform and remained on it for 30 s. If 60 s passed and the rats did not find the platform, they were gently guided to the platform. After each session, the rats were returned to their cages for resting. The time latency to reach the platform was recorded by a video tracking system. On day 5, to conduct a probe trial, platform was elevated above the water surface and time spent by the rats in the target quadrant (Q1) where platform was located, was recorded on days 1–4 (Gray et al., 2003 ▶).

**Measurement of serum antioxidant capacity**

Three solutions were used to measure serum antioxidant level: Solution 1 consisted of 1.5 ml of sodium acetate and 8 ml of concentrated acetic acid, diluted to 500 mL with distilled water; solution 2 consisted of 270 mg of iron (III) chloride, dissolved in 50 ml of distilled water and solution 3 consisted of 47 mg of Treeazin, dissolved in 40 ml ofHCl. Working solution was prepared by mixing 10 ml of solution 1, 1 ml of solution 2 and 1 ml of solution 3. Thereafter, 25 μl of serum samples was added to 5.1 ml of the working solution. The resulting mixture was incubated at 37°C for 15 min; then, the absorbance was measured at 593 nm wavelength (Rabiei et al., 2014 ▶).

**Measurement of serum malondialdehyde (MDA) level**

Briefly, 50 μL of the sera was mixed with 50 μL of 0.05% BHT, 400 μL of 0.44 M H_3_PO_4_ and 100 μL of 42 Mm TBA. The mixture was vortexed and then, heated in a boiling water bath for 1 hr. After cooling at 0℃ for 5 min, 250 μL of n-butanol was added to the mixture, vortexed, and then, centrifuged at 14000 rpm for 5 min. The supernatant absorbance was measured at 532 nm wavelength (Biondo et al., 2014 ▶; Rabiei et al., 2014 ▶) 

**Statistical analysis**

SPSS 18 was used to conduct data analysis. All data were expressed as mean± SD. One-way ANOVA followed by Duncan's test was used to compare the means among experimental groups. A p<0.05 was considered statically significant.

## Results

**Standardization of** ***B. vulgaris*** **leaf extract**

To standardize the plant extract, total phenolic flavonoid and flavonol compounds present in *B. vulgaris* leaf extract were measured. Total phenolic content of *B*. *vulgaris* leaf extract was 51 mg/g GAE expressed as mg phenol/g of dry matter. Total flavonoid and flavonolic contents were 17.3 mg/g and 2.5 mg/g, respectively, expressed as mg rutin equivalent/g of dry matter. The percentage of DPPH radical scavenging of extract is shown in Table 1. 

**Table 1:** DPPH radical scavenging activities for various concentrations of *Beta vulgaris* leaf extract

**samples**	**concentration**	**% of DPPH radical scavenging**
***Beta vulgaris leaf extract***	10	10.39
	20	18.01
	50	21.93
	100	39.95(IC50)
	250	81.52

**Morris Water Maze Test**

**Figure 1:**
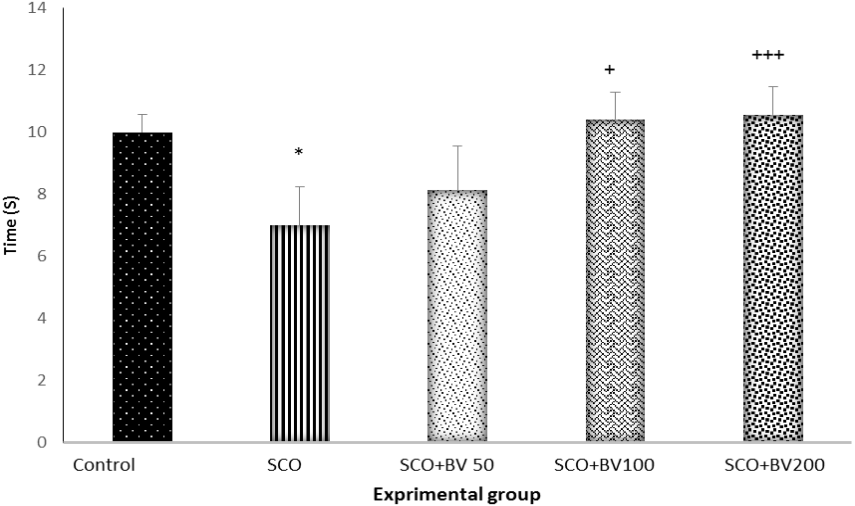
The time spent in target quadrant during the probe trial; the data are expressed as mean ± SD; n = 10 in each group; SCO+BV= scopolamine+ *B. vulgaris.* * shows significant difference compared to control group (*P<0.05); + shows significant difference compared to scopolamine group (+++p<0.001; +P<0.05

The time spent in the target quadrant during the 60-sec probe trial is illustrated in Figure 1. The control group spent significantly more time in the target quadrant (when the hidden platform was there during the previous days) compared to the scopolamine-treated group (p<0.05). Administration of* B. vulgaris* leaf extract at 100 and 200 mg/kg doses significantly increased the time spent in target quadrant in comparison with the scopolamine group (p<0.05).

**Figure 2:**
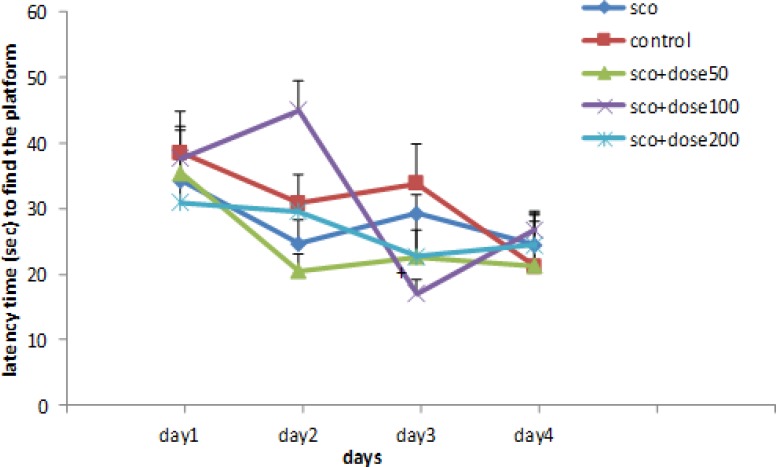
Comparison of latency time (sec) to find the platform among different experimental groups; the data are expressed as mean ± SD; n = 10 in each group; SCO+BV= scopolamine+ *B. vulgaris.* + Shows significant difference compared to scopolamine group (+P<0.05

The latencies to reach the platform during 4 consecutive days are shown in Figure 2. In the control group, the latency to find the platform during 4 consecutive days were shorter than scopolamine-treated group yet insignificantly. Treatment with *B. vulgaris* leaf extract at doses of 50, 100 and 200 mg/kg resulted in shorter latency time compared to the scopolamine group during the study period yet insignificantly. In rats treated with *B. vulgaris* extract at 200mg/kg, the latency to reach the platform on day 3 was significantly lower than that in scopolamine-treated rats (p<0.05).

**Effect of** ***B. vulgaris*** **leaf extract** **on serum antioxidant capacity**

**Figure 3:**
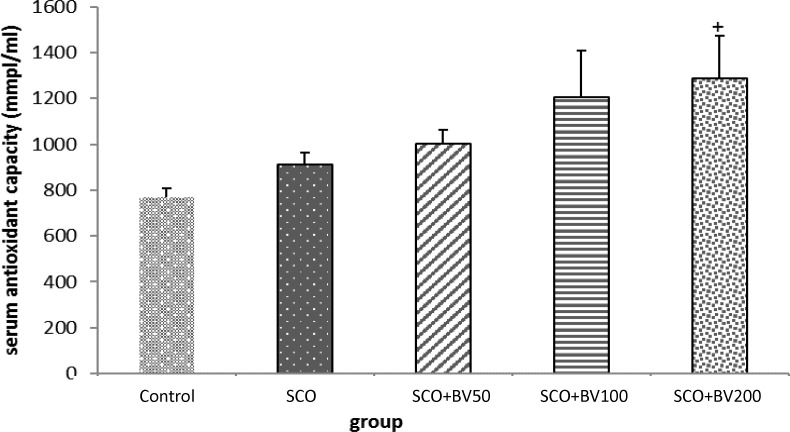
The effect of *Beta vulgaris* leaf extract on serum antioxidant capacity; the data are expressed as mean ± SD; n = 10 in each group; SCO+BV= scopolamine+ B*. vulgaris*;^+^ shows significant difference compared to scopolamine group

As Figure 3 illustrates, there was no significant difference in serum antioxidant capacity between control and scopolamine-treated rats. Administration of *B. vulgaris *leaf extract at concentrations of 50, 100 and 200 mg/kg to scopolamine-treated rats, increased serum antioxidant capacity, and the difference was significant at the dose of 200 mg/kg compared to the scopolamine group (p<0.05).

**Effect of** ***B. vulgaris*** **leaf extract** **on serum MDA level**

**Figure 4:**
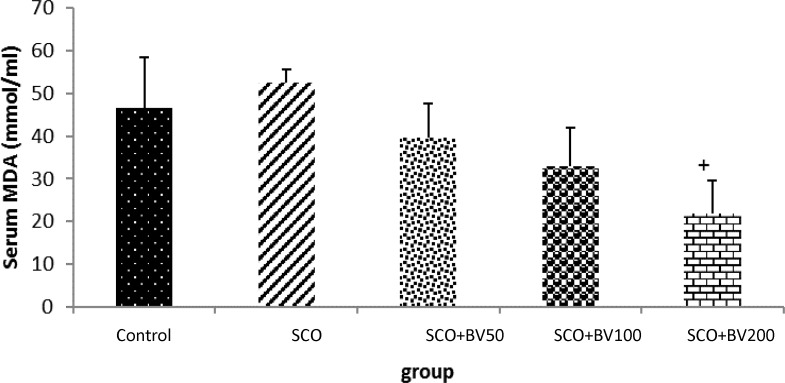
The effect of *Beta vulgaris *leaf extract on serum MDA level in rats; the data are expressed as mean ± SD; n = 10 in each group; SCO+BV= scopolamine+ *B. vulgaris*; ^+^ shows significant difference compared to scopolamine group

The effect of *B. vulgaris *leaf extract on serum MDA level in rats is shown in Figure 4. The scopolamine-treated rats presented higher serum MDA levels when compared to control group (p>0.05). Administration of *B. vulgaris *leaf extract 50, 100 and 200 mg/kg decreased serum MDA level in comparison with scopolamine group, and 200 mg/kg of *B. vulgaris *leaf extract caused the lowest serum MDA level with a significant difference compared to other concentrations.

## Discussion

The results of the present study indicated that scopolamine led to learning and memory impairments in rats as assessed by Morris water maze test. This result is in agreement with the previous studies (Chuong et al., 2014 ▶; Rabiei et al., 2015 ▶). Scopolamine is considered a cholinergic antagonist which interferes with the transmission of acetylcholine in the central nervous system leading to memory deficits (Misane et al., 2003 ▶).

The results also indicated that the treatment of scopolamine-treated rats with *B. vulgaris* leaf extract at 100 and 200 mg/kg significantly increased the time spent in target quadrant of Morris water maze (p<0.05). *B. vulgaris* extract (100 mg/kg) also significantly increased latency to reach the platform on day 3. These results showed the protective effects of *B. vulgaris* extract on scopolamine-induced memory deficits. 

The results of the present study showed that scopolamine resulted in reduction of serum antioxidant capacity, but it increased serum MDA levels yet insignificantly. These results are in agreement with studies that reported increased levels of MDA after treatment with scopolamine (Rabiei et al., 2015 ▶; Wilson, 1997 ▶). Increase in MDA, the most well-known secondary product of lipid peroxidation, is a valid marker for *in vivo* lipid peroxidation and can be used to determine the severity of oxidative damage to cells and tissues (Grotto et al., 2009 ▶)

In our study, administration of *B. vulgaris *leaf extract at 200 mg/kg significantly increased the antioxidant capacity and decreased serum MDA levels compared to scopolamine group. Jain et al. (2012) ▶ also reported that treatment with *B. vulgaris* leaf extract significantly decreased hepatic MDA level and increased GSH, suggesting that the antioxidant effect of *B. vulgaris* leaf extract plays an important protective role against ethanol-mediated toxicity (Jain et al., 2012 ▶)*. B. vulgaris* leaf also could improve antioxidant status in mice fed with high-fat diet (Jain et al., 2012 ▶). The results of this study indicated that *B. vulgaris *leaf extract exhibited protective activity against the adverse effects of scopolamine; therefore, it could reduce the brain damage and improve brain and memory activities in scopolamine-treated rats. These findings may be associated with antioxidant activity of *B. vulgaris *leaf extract and inhibition of oxidative stress in the rats' brains.

In the present study, we observed that *B. vulgaris* leaf extract at 100 µg/ml could inhibit 39.95% of DPPH radicals that reached 81% when the dose of 250 µg/ml of this extract was administered. These results indicate remarkable antioxidant and free radical-scavenging activity of *B. vulgaris* leaf extract which is comparable to those of other antioxidant agents. The free radical-scavenging activity of *B*. *vulgaris* leaf extract can be due to the presence of bioactive compounds with antioxidant properties such as polyphenols, flavonoids, and vitamin C. The total phenolic content in this plant was 51 mg GAE/1 g of the dried extract. Jain et al., (2012) ▶ reported that the main constituents of *B. vulgaris* leaf are sterols, triterpenoids, phenols, tannins, flavonoids, alkaloids, glycosides, and saponins (Jain et al., 2012 ▶). Polyphenols, flavonoids, saponins, glycolipid, phospholipids, fatty acids, folic acid, and ascorbic acid are some of the major components of *B. vulgaris* leaf extract that give antioxidant property to this extract. In addition, folic acid, iron, calcium, phosphorus, zinc, and vitamins A, B, and C can play important roles in brain development and motor function (Bolkent et al., 2007 ▶; Mroczek et al., 2012 ▶; Mokhtari-Dehkordi et al., 2014 ▶). Sulakhiya et al., (2016) ▶ reported that *B. vulgaris* leaf extract had desirable effect on depression which was attributed to the high levels of antioxidant compounds such as polyphenols (betalains and betaine), flavonoids, and vitamin C in *B. vulgaris *leaf. 

The protective effect of *B. vulgaris *leaf extract on scopolamine-induced memory impairment may be related to increases in acetylcholine levels in the brain. We found no previous study on cholinergic effect of *B. vulgaris *leaf extract; therefore, it is recommended to further evaluate the cholinergic effect of *B. vulgaris* extract.

The results of this study indicated that *B. vulgaris *leaf extract exhibited protective activity against scopolamine-induced brain and memory impairments. This effect may be associated with increased activity of antioxidant defense system and inhibition of oxidative stress in the brains of rats. *B. vulgaris* leaf extract can be used as a beneficial medicinal herb for improving brain and memory complications due to high levels of polyphenolic antioxidant compounds.

## Conflict of interest

The authors have no conflicts of interest to declare.
